# One Odontogenic Cell-Population Contributes to the Development of the Mouse Incisors and of the Oral Vestibule

**DOI:** 10.1371/journal.pone.0162523

**Published:** 2016-09-09

**Authors:** Maria Hovorakova, Katerina Lochovska, Oldrich Zahradnicek, Kristina Domonkosova Tibenska, Michaela Dornhoferova, Lucie Horakova-Smrckova, Silvia Bodorikova

**Affiliations:** 1 Department of Teratology, Institute of Experimental Medicine, Academy of Sciences of the Czech Republic, 14220, Prague 4, Czech Republic; 2 Department of Anthropology and Human Genetics, Faculty of Science, Charles University, Prague, Czech Republic; 3 Department of Anthropology, Faculty of Natural Sciences, Comenius University in Bratislava, Bratislava, Slovak Republic; 4 Department of Cell Biology, Faculty of Science, Charles University, Prague, Czech Republic; University of Texas at Austin Dell Medical School, UNITED STATES

## Abstract

The area of the oral vestibule is often a place where pathologies appear (e.g., peripheral odontomas). The origin of these pathologies is not fully understood. In the present study, we traced a cell population expressing Sonic hedgehog (*Shh*) from the beginning of tooth development using Cre-LoxP system in the lower jaw of wild-type (WT) mice. We focused on *Shh* expression in the area of the early appearing rudimentary incisor germs located anteriorly to the prospective incisors. The localization of the labelled cells in the incisor germs and also in the inner epithelial layer of the vestibular anlage showed that the first very early developmental events in the lower incisor area are common to the *vestibulum oris* and the prospective incisor primordia in mice. Scanning electron microscopic analysis of human historical tooth-like structures found in the vestibular area of jaws confirmed their relation to teeth and thus the capability of the vestibular tissue to form teeth. The location of labelled cells descendant of the early appearing *Shh* expression domain related to the rudimentary incisor anlage not only in the rudimentary and functional incisor germs but also in the externally located anlage of the oral vestibule documented the odontogenic potential of the vestibular epithelium. This potential can be awakened under pathological conditions and become a source of pathologies in the vestibular area.

## Introduction

The area located externally to the dentition and lined by the gums, lips and cheeks in humans is called the oral vestibule or “*vestibulum oris*”. This area is often a place where pathologies appear (e.g., peripheral odontomas, dentigerous cysts). A peripheral odontoma can be associated with the presence of non-erupted [1–3] or erupted tooth-like structures (e.g. [4]. The question arises, how dental anomalies develop in an area where the dental lamina is not present. Previously, the origin of these pathologies has been explained by soft tissue remnants of the odontogenic epithelium such as gingival rests of Serres [3, 5] or by the pathological development of the tooth germ of an absent tooth in a tooth-row [4].

Surprisingly, in contrast to the generally accepted concept of a continuous vestibular lamina giving rise to the oral vestibule in humans, the human oral vestibule has been documented on computer-aided 3D reconstructions originating from a series of discontinuously oriented epithelial bulges and ridges developing in a close relationship to the dental epithelium [6, 7]. In mice, the incisive and diastema regions of the oral vestibule develop from the vestibular lamina in the lip region (lip-furrow band). In the cheek region, the cheek-furrow gives rise to the distal part of the oral vestibule [8].

In a mouse model of odontogenesis, the expression of Sonic hedgehog (*Shh*) is limited to the odontogenic areas in embryonic mouse jaws and it is considered to be the marker of early odontogenesis. *Shh* is not expressed in the vestibular epithelium itself but an early anterior expression of *Shh* has been found anteriorly to the incisor germs at embryonic day (E) 12.5 preceding the signaling center of a functional incisor appearing as late as at E13.5 in both the upper and lower jaws. This early superficial expression domain has been associated with prelacteal rudimentary tooth formation anteriorly to the prospective mouse incisor. It has been suggested that its cells could also contribute to the vestibular epithelium [9, 10].

Using a Cre-loxP transgenic system, we traced the fate of a cell population expressing *Shh* anteriorly to the lower prospective functional incisors in the area of rudimentary incisor appearance. Labelled cells originating in this one cell population were found not only in the rudimentary and functional incisor primordium, but also in the externally located anlage of the oral vestibule, thus documenting the feasible odontogenic potential of the vestibular epithelium. Scanning electron microscopic (SEM) analysis of tooth-like structures found externally to the dental arch in a historical human skull confirmed their relation to teeth and it supported the results obtained in the mouse model that the vestibular epithelium may keep its odontogenic potential and thus the capability to form teeth. It demonstrated the formation of vestibular teeth in humans.

Based on this, we show in the present study that the first very early developmental events in the lower incisor area are common to the oral vestibule and the prospective incisors primordia in mice. The original odontogenic potential of epithelial cells in the anlage of the oral vestibule can be awakened under pathological conditions, and its abnormal development can lead to the formation of dental pathologies in the area external to dentition. This finding is also essential for explaining the developmental background of pathologies with dental tissue presence (e.g., odontomas) located in the vestibular area in humans and other mammals.

## Material and Methods

### Mouse Embryos

Cre-loxP system Nr.1: The C57BL/6 mouse strain carrying the fusion protein Shh-EGFP (Enhanced Green Fluorescent Protein) and Cre recombinase from the endogenous *Shh* locus (B6.Cg-*Shh*^*tm1(EGFP/cre)Cjt*^/J [11]) and Cre recombinase sensitive transgenic mice containing *LacZ* (beta-galactosidase) inserted into the *Gt(ROSA)26Sor* locus [12] were reciprocally crossed in order to mark all cells expressing *Shh* and all their descendants until the time of harvesting of embryos (Table 1).

**Table 1:** Table of the used material—Cre-loxP system Nr.1. Numbers of harvested embryos (obtained using the Cre-loxP system Nr.1: LacZ x B6.Cg-*Shh*^tm1(EGFP/cre^)Cjt/J) positive for X-gal staining.

E	Number of specimens
12.5	4
13.5	6
14.5	4
15.5	12
16.5	4

Cre-loxP system Nr.2: C57BL/6 mice carrying tamoxifen-inducible Cre fused with the *Shh* allele (B6.129S6-Shh<tm2(cre/ERT2)Cjt>/J [11]) and Cre recombinase-sensitive transgenic mice containing *LacZ* (beta-galactosidase) inserted into the *Gt(ROSA)26Sor* locus were used for the cell tracing study. The strains were reciprocally crossed in order to mark the cell population expressing *Shh* only from the time of the tamoxifen injection into pregnant female mice to create a narrow developmental window showing the distribution of labelled cells after the tamoxifen injection in the vestibular epithelium (Table 2).

**Table 2:** Table of the used material—Cre-loxP system Nr.2. Numbers of harvested embryos (obtained using the tamoxifen inducible Cre-loxP system Nr.2: LacZ x B6.129S6-Shh<tm2(cre/ERT2)Cjt>/J) 24/48/72/96 and 120 hours (h) after tamoxifen injection at embryonic day (E)11.5 positive for X-gal staining.

Time	Tamoxifen/E11.5
24h	3
48h	4
72h	10
96h	4
120h	4

The breeding pairs of all transgenic mouse strains were purchased from the Jackson Laboratory (Maine, USA). The mice were genotyped using the Jackson Laboratory’s protocols.

The appropriate mice were mated overnight and the midnight before the morning detection of a vaginal plug was regarded as E0.0. The embryos were harvested at E12.5–16.5 for *Shh* expressing cell lineage tracing using Cre-loxP system Nr.1 (see Table 1) and 24–120 hours after tamoxifen injection for the tracing of cells expressing *Shh* in the anterior rudimentary incisor anlage using the tamoxifen inducible Cre-loxP system Nr.2 (see Table 2).

The pregnant mice were sacrificed by cervical dislocation to minimize suffering and after that the embryos were removed from the uterus. Immediately after removing the embryos, their wet body weights were determined [10, 13]. All embryos were processed individually.

All used animals were fed and watered ad libitum. Housing of animals and in vivo experiments were carried out in strict accordance with the national and international guidelines (ID 39/2009). This study was performed under supervision of the Professional committee for guarantee of good life-conditions of experimental animals at the Institute of Experimental Medicine, Academy of Sciences of the Czech Republic, Prague, Czech Republic and approved by the Expert Committee at the Academy of Sciences of the Czech Republic (permit number: 027/2011).

### Tamoxifen Administration and X-Gal Staining

Pregnant female mice of the Cre-loxP system Nr. 2 were injected intra-peritoneally with tamoxifen at E11.5, when *Shh* is expressed only in the anterior expression domain in the prelacteal incisor [9]. Tamoxifen was administrated in a dose of 0.225 mg/g of body weight [14]. Such a concentration is not hazardous for pregnant mice or embryos and is sufficient for the fast activation of recombination. The embryos were harvested 24/48/72/96 and 120 hours after tamoxifen application by intraperitoneal injection into pregnant female mice on embryonic day (E) 11.5. The heads of embryos were washed in a phosphate buffer at 4°C and pre-fixed for 20 minutes in 4% paraformaldehyde (PFA). The samples were stained in the staining phosphate buffer (X-gal (Sigma) concentration 3mM) and beta-galactosidase activity was detected by incubation in the dark during night at 37°C. Samples with positive staining (see Tables 1 and 2) were post-fixed in PFA (4%) overnight. After post-fixation, the samples were washed in PBS. The upper and lower jaws were dissected, and the lower jaws were photographed using a Leica MZ6 stereomicroscope equipped with a Leica EC3 digital camera. After photo-documentation, the samples were post-fixed in Bouin solution for a minimum of two weeks, and then histologically processed.

### Histology of X-Gal Stained Samples

The X-gal positive samples were routinely embedded in paraffin and 10μm thick serial frontal sections were prepared. The sections were counterstained with Fast red (Fluka). The stained sections were dehydrated and covered using Neomount (Merck).

### Dissociation of Epithelium

The lower incisor area of an embryo at ED14.5 72 hours after tamoxifen administration (Cre-loxP system Nr.2) was dissected and washed in PBS. For better visualization of labelled areas, the dental and adjacent oral epithelium was dissociated using dispase by incubating in 37°C for 20 minutes. The epithelium was transferred into PBS and fixed in PFA (4%) for 15 minutes in RT. The X-gal staining was performed according to the standard protocol (see above: Tamoxifen administration and X-gal staining).

### Whole Mount In Situ Hybridization

To visualize the early odontogenic areas in the lower mouse incisor region, *Shh* expression was detected using WISH in 8 CD1 mouse embryos. Mandibles were dissected at ED12.5 (body-weights 67–110mg), washed in Rnase free PBS (pH 7.4), and fixed in PFA (4%) over night at 41C. Specimens were hybridized according to a standard protocol. The probe for *Shh* was generated by in vitro transcription from cDNA fragment (kind gift from Dr. A. McMahon, Harvard University, Cambridge, MA). The hybridized samples were documented using a Leica MZ6 stereomicroscope connected to a DC480 digital camera Leica (Leica Microsystems GmbH).

### Processing of a Lower Jaw of a Mangrove Monitor Lizard (*Varanus indicus*)

The egg was obtained from the Prague Zoological Garden and incubated at 28°C. According to the Czech National Board Law for Animal Protection and against Cruelty to Animals (ID 246/1992) the ethical approval is not required for working with reptilian embryos obtained from eggs. The embryo was harvested 56 days after oviposition. It was anesthetized in MS222 and after that decapitated and fixed in 4% PFA for 2 days, dehydrated through a series of ethanol and embedded in paraffin. The head was frontally sectioned at 10μm and the sections were stained by haematoxylin, eosin and alcian blue for the visualization of the tissue structures.

### Historical Material for Scanning Microscopy

At the Gan locality (Galanta district, Slovak Republic), a rescue archaeological excavation took place in the years 2007 and 2008. All necessary permits were obtained for the described study, which complied with all relevant regulations. Archaeological research in the building area of "The logistic center and light manufacturing Gan" was permitted by The Monuments Board of the Slovak Republic—regional office in Trnava (Statement no. TT-06/738/2076 / Ho). The statement was issued under Act No. 49/2002 Z. z. (Act on the protection of monuments and historic sites). Archaeological excavation was realized by the Homeland Museum in Galanta (Slovakia) and Homeland Museum in Hlohovec (Slovakia).

A burial site named Gan A has a special importance, because it is the third largest cemetery dated to the Migration period (5th - 6th c. AD) in the Slovak Republic. Thirty-nine graves were excavated containing the skeletal remains of 30 individuals (17 adults and 13 subadults). Skeletal remains are deposited at the Department of Anthropology, Faculty of Natural Sciences, Comenius University in Bratislava, Slovak Republic.

In the grave AH19, a female of age 60+ was buried. In the skull, 23 teeth were present, two teeth were lost during her life, four teeth (all upper incisors) were lost *post mortem*, both lower third molars were not erupted, and the right posterior part of the maxilla was damaged (not available). Non-dental degenerative pathological changes to the skeleton were detected: Schmorl´s nodes and spondylosis of the vertebrae, which was most significant in the third and fourth lumbar vertebrae [15].

Three tooth-like structures were found in this female. Two of them were found in the mandible, located externally to the alveoli. The smaller dental particle was present on the right side, and was located under the *septum alveolarium* between the premolars. The second, larger particle was found on the left side and was located under the second premolar. The third particle was found among the fragments of facial bones and could have originated from the maxilla.

For surface observation, the larger dental particle was extracted from the mandibular bone and, together with the small free dental particle, were photographed using a Canon EOS 650D camera equipped with a Canon Macro Lens EF 100mm, 1:2.8 L IS USM objective.

### Scanning Electron Microscopy (SEM)

The two denticles were cleaned in acetone, than in an ultrasonic bath and dried. Finally, the samples were coated with gold for scanning microscopy. The samples were examined using SEM JEOL 6380LV scanning electron microscope at the Laboratory of Electron Microscopy, Faculty of Science, Charles University in Prague (Czech Republic).

## Results

**Fig 1:**
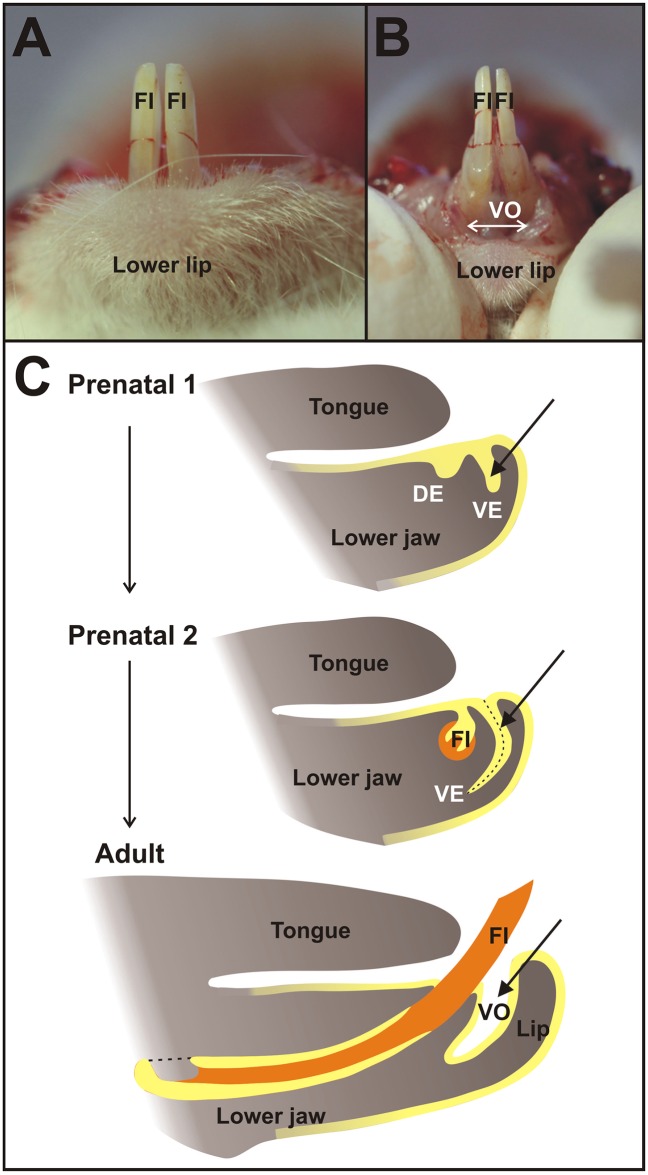
Oral vestibule in the mouse. In the mouse lower jaw (**A**), the anterior lower oral vestibule (VO) is a free space of oral cavity bounded externally (labial) by the mucosa of the lips and orally by the alveolar mucosa, gingiva, and teeth (**B, C**). It originates as a vestibular lamina (VE) adjacent to the dental epithelium (DE), which is a developmental base of functional incisors (FI) comprising epithelial (yellow) and mesenchymal (orange) material. Vestibular lamina itself gives rise to the epithelial lining of the oral vestibule space in an adult mouse lower jaw.

**Fig 2:**
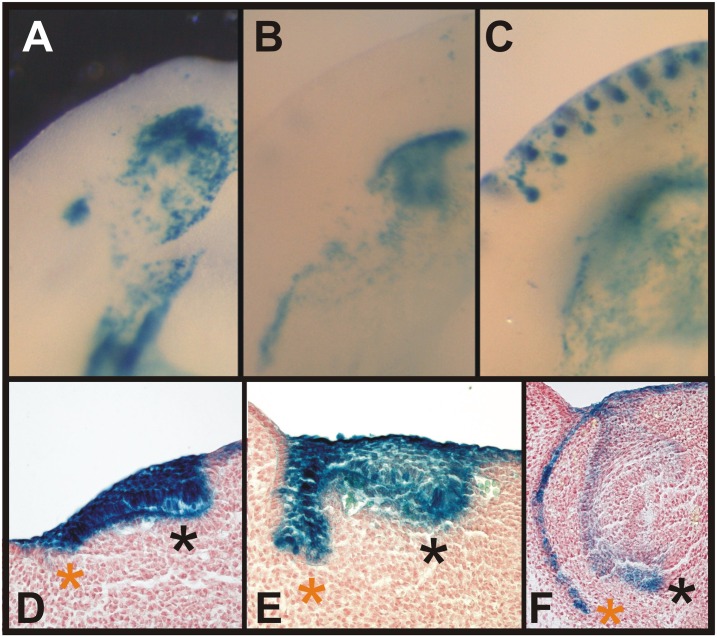
Descendants of the cells originally expressing *Shh* are located in the lower vestibular anlage. Embryonic jaws (obtained using the Cre-loxP system Nr.1 - see Methods) with permanently stained (blue) cells showed the descendant lineage of cells first expressing *Shh* (**A-C**). Histological sections (**D-F**) revealed that the blue labelled cells were localized in the dental epithelium (black asterisk) as well as in the epithelial anlage of the oral vestibule (orange asterisk) at E12.5 (**A, D**), 13.5 (**B, E**) and 14.5 (**C, F**). The blue labelled cells were finally concentrated in the inner epithelial layer of the vestibular anlage (**F**) confirming the contribution of cells expressing *Shh* in the early domain of the rudimentary incisor to this epithelial structure.

**Fig 3:**
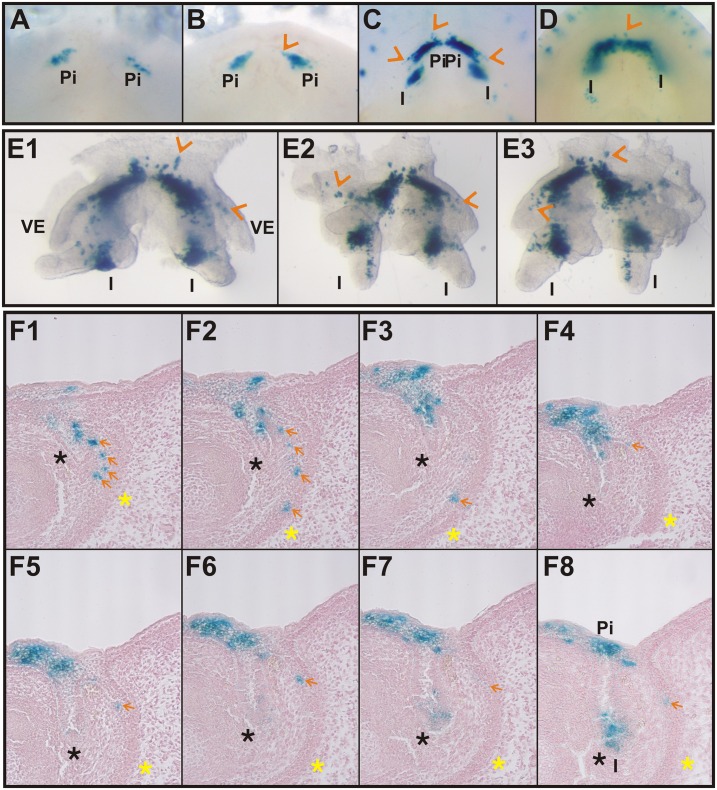
Cells expressing *Shh* in the early rudimentary incisor anlage head towards the oral vestibule. Embryonic jaws (obtained using the Cre-loxP system Nr.2 –see Methods) with permanently stained (blue) cells showed the descendant lineage of cells expressing *Shh* after an intraperitoneal tamoxifen injection started recombination at E11.5 (tamoxifen inducible Cre-loxP system), at which time the presence of only the first anterior superficial *Shh* expression correlated with rudimentary incisor (pi) formation was apparent [9] (**A-D**). Only one area of blue labelled cells was present in embryos at the early stages of development (E12.5-**A**, E13.5-**B**) representing only the descendants of the firstly appearing superficial *Shh* expression located in the area of rudimentary incisor formation [9]. Two areas of blue labelled cells were present at E14.5 (**C, E1-E3**) and 15.5 (**D**), the anterior one related to the rudimentary prelacteal incisor and the posterior one related to the functional incisor (I). Frontal histological serial sections (**F1-F8**) in the specimen at E15.5 after tamoxifen injection at E11.5 revealed that blue labelled cells were localized not only in the dental epithelium (black asterisk) but also in the epithelial anlage of the oral vestibule (yellow asterisk) in its inner epithelial layer (orange arrows) confirming the contribution of *Shh* expressing cells from the first anterior *Shh* expression domain of the rudimentary incisor anlage to the vestibular epithelium (VE).

The anterior oral vestibule in the mouse incisor region is a free space bounded externally (labial) by the mucosa of the lips and orally by the alveolar and gingival mucosa, and teeth (Fig 1). Using two transgenic Cre-loxP systems: ((1) B6.Cg-*Shh*^tm1(EGFP/cre^)Cjt/J mice crossed with Cre recombinase-sensitive transgenic mice containing *LacZ* (Fig 2) and (2) B6.129S6-*Shh*<tm2(cre/ERT2)Cjt>/J mice possessing tamoxifen inducible Cre crossed with Cre recombinase sensitive transgenic mice containing *LacZ*, Fig 3), we traced the cells expressing *Shh* during early lower incisor development and found their descendants in the epithelial anlage of the oral vestibule located externally to the anlage of the dentition (Figs 2 and 3).

### Descendants of the Cells Originally Expressing *Shh* Are Localized in the Lower Vestibular Anlage

The first Cre-loxP system set up by reciprocal crossing of two mouse strains, B6.Cg-*Shh*^tm1(EGFP/cre^)Cjt/J mice and Cre recombinase-sensitive transgenic mice containing *LacZ*, allowed the tracing of all the cells that firstly expressed *Shh* from the absolute beginning of the embryonic development as well as all of their descendants in the offspring. The cells were permanently stained (blue) by X-gal staining (Fig 2A–2F). We observed the localization of blue labelled cells in both locations in the center of interest: the incisor primordia as well as in the epithelial anlage of the oral vestibule from E12.5 till 14.5 (Fig 2D–2F, black and red asterisk, resp.). Interestingly, the blue labelled cells were concentrated in the inner epithelial layer of the vestibular anlage (Fig 2F, red asterisk). The outer epithelial layer was negative. The localization of blue cells in the vestibular anlage confirmed the general contribution of *Shh* expressing cells to this epithelial structure.

### Cells Expressing *Shh* in the Early Rudimentary Incisor Anlage Head towards the Oral Vestibule

The presence of blue cells in the vestibular epithelium evoked a question, how the cells that express *Shh* early are distributed during the development of the lower incisor area. To clarify the occurrence of positive cells in the vestibular anlage, a new Cre-loxP system was created and used to mark and follow the fate the cell population expressing *Shh* during a narrow time period (applications of tamoxifen at E11.5–12.5). This is the period, when the *Shh* expression domain related to rudimentary incisor development is appearing anteriorly to the prospective lower functional incisor germ [9].

To determine the contribution of cells expressing *Shh* during the early development of the incisor germ to the vestibular anlage, B6.129S6-*Shh*<tm2(cre/ERT2)Cjt>/J mice with the tamoxifen inducible Cre fused with *Shh* in the endogenous locus were reciprocally crossed with Cre recombinase sensitive transgenic mice containing *LacZ*. Embryos were used to mark and follow the fate of the cells appearing in the early rudimentary *Shh* expression domain (tamoxifen application at E11.5) located anteriorly to the functional incisor germs (Table 1).

The tamoxifen injection induced recombination in the cells actively expressing *Shh* from the related stage of development (see S1 and S2 Figs). X-gal staining permanently labelled all cells with positive beta-galactosidase activity and also all of their descendants. Induction at E11.5 showed the presence of labelled cells in the basal part of the oral epithelium anteriorly to the prospective functional incisor primordium after 24/48 hours at E12.5/13.5 (Fig 3A and 3B), as well as within the functional incisor germ after 72 hours at E14.5 and after 96 hours at E15.5 (Fig 3C and 3D). However, blue labelled cells were localized not only in the tooth primordia. They were also present in the anlage of the oral vestibule (Fig 3E1–3E3). Their position corresponded to the inner epithelial layer of the vestibular anlage (Fig 3F1–3F8).

The localization of positive labelled cells in the vestibular epithelium throughout the whole period of observation following tamoxifen induction of recombination at E11.5 signified that these labelled cells originated in the early *Shh* expression domain of the epithelial anlage corresponding to the rudimentary incisor located anteriorly to the lower functional incisor. This detection confirmed the contribution of odontogenic cells (originating in the odontogenic tissue of rudimentary incisor anlage) to the anlage of the oral vestibule in the mouse mandible.

### Tooth-Like Structures External to the Mandibular Tooth Alveoli Found in a Historical Skull Confirm the Odontogenic Potential of the Epithelium Externally to the Dentition

**Fig 4:**
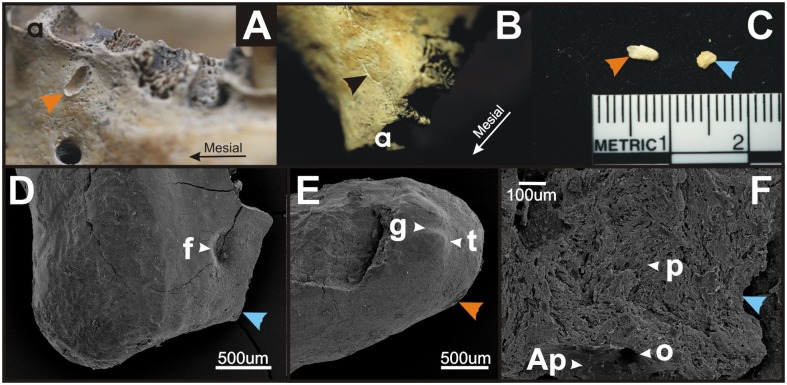
Tooth-like structures in a historical skull confirm the odontogenic potential of the vestibular epithelium. During rescue archaeological research in 2007 and 2008 at the Gan locality (Galanta district, Slovak Republic, the Migration Period, 5th - 6th c. AD), three tooth-like structures were found in a female skull excavated from grave AH19 (**A-F**). Two tooth-like structures were located in the mandible (**A-B**) externally to the alveoli (a), one on the surface of the mandibular bone (orange arrowhead, **A, C, E**) and one within the mandibular bone (black arrowhead, **B**). One dental particle was found free among the bone fragments (blue arrowhead, **C, D, F**). Scanning electron microscopy (**D-F**) showed aprismatic (Ap) and prismatic enamel (p) with tubercles (t) and fossae (f) or grooves (g) formation on the surface of the scanned denticles. Openings (o) presumably for vessels or nerves were detected.

Two tooth-like structures were found in the mandible of a female from grave AH19 in Gan locality in the Slovak Republic (Fig 4A–4F). These tooth-like structures were located outside the dental arch externally to the alveoli of teeth (Fig 4A and 4B). Their location corresponded with the demarcation of vestibular area.

Using scanning electron microscopy, we found that the tooth-like structures showed characteristics of dental tissue (Fig 4D–4F) supporting their relation to teeth: these denticles presented aprismatic and prismatic enamel with tubercles and depressions or grooves formation (Fig 4D–4F). Openings presumably for vessels or nerves were also detected in these denticles (Fig 4F).

### The Morphological Similarity of the Human Vestibular Epithelium and Tooth Gland Lamina in Reptiles

**Fig 5:**
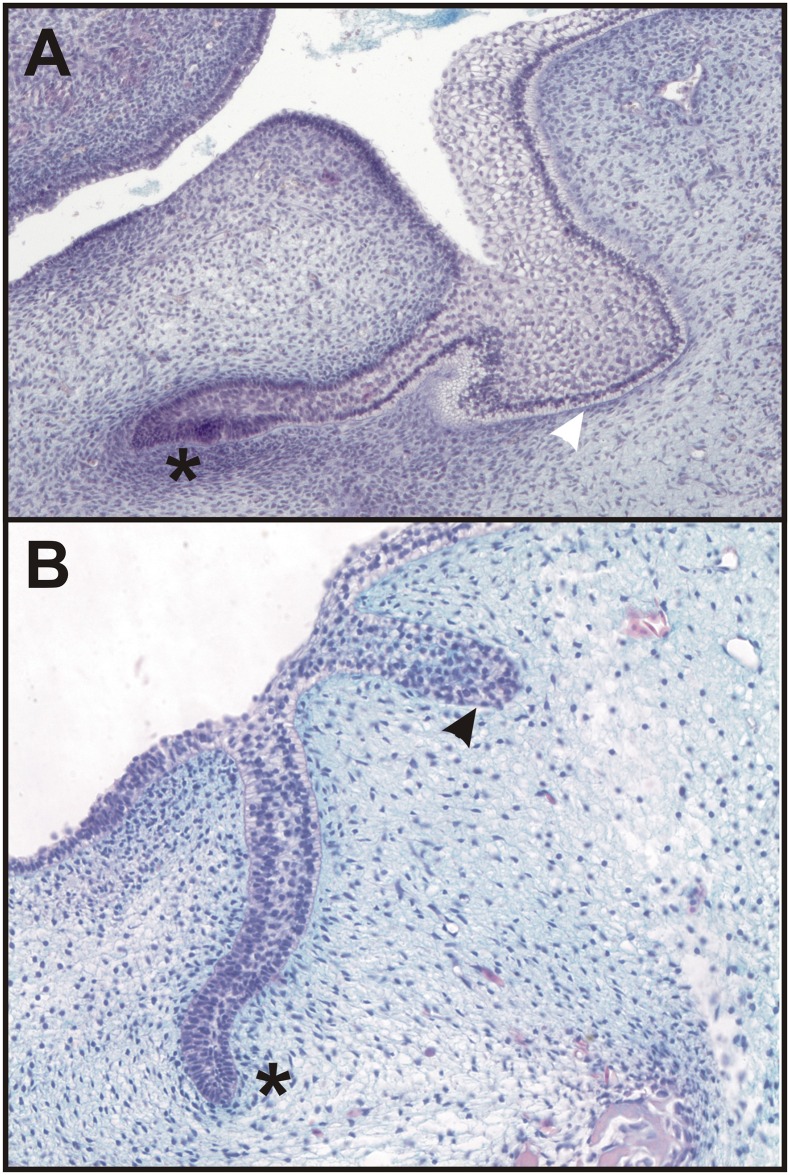
The morphological similarity of the human vestibular epithelium and tooth gland lamina in reptiles. In humans, the oral vestibule originates in the epithelial structures (white arrowhead) located externally to the dental epithelium (black asterisk) (**A**) discontinuously arranged in a series externally to the dental mound (lower jaw on a frontal histological section of the head of a human embryo from the archive of Dr. Hovorakova [6, 7]. In some reptiles (shown in a mangrove monitor lizard—*Varanus indicus*), small glands located externally to individual teeth originate from an epithelial tooth gland lamina (black arrowhead) (**B**).

In humans, the oral vestibule originates in the discontinuously arranged epithelial bulges and ridges running in a series externally to the dental lamina (Fig 5A). In some reptile species (eg. mangrove monitor lizard—*Varanus indicus*), small glands located externally to individual teeth originate from an epithelial tooth gland lamina (Fig 5B), which is adjacent externally to the dental lamina. The histology of the human vestibular epithelium and the tooth gland lamina of a mangrove monitor lizard revealed an unexpected morphological similarity supporting their close relationship (compare Fig 5A with 5B).

## Discussion

The oral vestibule is the portion of the oral cavity bounded on one side by the teeth and gingivae, or the residual alveolar ridges, and on the other side by the lips (labial vestibule) and cheeks (buccal vestibule). In humans, the oral vestibule originates in the discontinuously arranged epithelial bulges and ridges running in a series externally to the dental lamina (Fig 5A, [6, 7]. The epithelial structures located externally to the developing dentition were observed by scientists more than hundred years ago and correlated with “prelacteal dentition” [16, 17] or the tooth glands of reptiles [17].

In reptiles, the movable lips, cheeks and oral vestibule of mammalian type, in the accepted sense, do not occur. The epithelial tooth gland lamina (Fig 5B), which is adjacent externally to the dental lamina [18] gives rise to glands moistening the oral cavity with their secretion in distinct reptilian species. The close association of the dental glands and the teeth in reptiles emphasizing the similarity of these structures during early development has been noted [19]. The morphological similarity of the human vestibular epithelium and the tooth gland lamina in some reptiles (eg. mangrove monitor lizard—*Varanus indicus*) is unexpected suggesting their possible developmental relationship (compare Fig 5A with 5B).

Interestingly, active *Shh* expression has not been detected in the vestibular epithelium of the lower jaw in E11.5–13.5 mouse embryos [9]. Contrary to this, the cell-lineage tracing used in the present study showed the localization of blue marked cells (descendants of the cells expressing *Shh* from the beginning of prenatal development) in the incisor primordia as well as in the anterior part of the epithelial anlage of the oral vestibule anteriorly to the incisors (see Fig 2). This observation evoked the question, why are the descendant cells of the *Shh* expressing cell lineage located in the vestibular epithelium if no cells in the vestibular anlage express *Shh*. This highlighted the possibility that these cells do not originate in the vestibular epithelium directly but in a different part of the oral cavity.

It has been documented using in situ hybridization that the early superficial *Shh* expression in the anterior part of the mouse jaw (from E11.5–13.5) corresponds to the rudimentary incisor primordium appearing as an epithelial budding preceding the functional incisor germ in both the upper and lower jaws in WT mice. Determining of a position and formation of the functional incisor tooth has been suggested as a presumable function of these cells early expressing *Shh*. The signaling center of the functional incisor appears as late as at E13.5, when the early anterior *Shh* expression disappears [9, 10]. In the present study, the distribution and fates of cells of the early *Shh* expression domain during the subsequent development of the incisor area was documented using tamoxifen inducible Cre fused with *Shh* in the endogenous locus. Recombination in the population of cells expressing *Shh* in the early *Shh* expression domain started by tamoxifen injection at E11.5 (which is the period of the early rudimentary *Shh* expression domain´s presence anteriorly to the functional incisor germ) revealed the blue labelled cells present in the functional incisor anlage but also the inner cell layer of the vestibular epithelium (Fig 3F1–3F8). The mixture of blue labelled and negative cells in the vestibular anlage documents dual developmental origin of this structure. Interrestingly, after the induction of recombination at ED14.5, when the early signaling center related to rudimentary incisor does not express *Shh* and only signaling center of functional incisor is active, the superficial area descendant of rudimentary incisor anlage as well as the area of the vestibular epithelium (yellow asterisk) remained blue cells negative (compare S3 Fig to Fig 3).

Our findings clearly showed that the cells expressing *Shh* in the early anterior and more superficially located expression domain contribute to the formation of the vestibular lamina (Fig 3F1–3F8). The blue labeling of the vestibular epithelia had to be result of the migration of cells into the vestibular epithelium originally descend from the early *Shh* expression domain present until E13.0 in the prospective incisor area. This supports the common developmental origin of incisors and of the inner vestibular cell layer. The outer vestibular cell layer might take its origin in the oral side of the lip and cheek mucosa.

The patients with Ellis-van Creveld syndrome exhibit pathologies in the vestibular area represented by multiple labial frenula [20]. It has been shown in Evc null mice that Sonic and Indian hedgehog signaling are disrupted in tooth and bone development. From this aspect, Ellis-van Creveld syndrome could confirm the involvement of cells originally expressing *Shh* in the vestibular anlage formation shown in the present study. The failure of *Shh* signaling would thus cause the defects in the patients with Ellis-van Creveld syndrome.

Pathologies connected with the dentition are often present in the vestibular area. In mice, odontogenic ameloblastomas have been documented in transgenic amel00 mice [21]. In humans, peripheral odontomas can be found in the area external to the dentition [1–4, 22, 23]. The developmental origin of odontomas is still unknown. The histogenesis of this type of odontoma has been associated with soft tissue remnants of the odontogenic epithelium such as the gingival rests of Serres, retaining the ability to pursue epithelial-mesenchymal interactions that could lead to odontoma formation [3, 5]. In other cases, the co-localization of odontomas with missing teeth in the dental arch has suggested that the origin of this pathology could be the abnormal development of the germ of a missing tooth [4].

A very rare case of peripheral odontoma with tooth-like structures erupted into oral cavity has been reported recently [4]. Histopathological analysis showed that the tooth-like structures were composed of enamel, dentin, pulp chamber and cementum in the same order of arrangement as that in a normal tooth. These structures were surrounded by a thin epithelium and embedded in dense fibrous connective tissue, demonstrating their peripheral origin. The rudimentary denticles that had erupted from the odontoma had a fragile insertion into the gingival tissue, which was clinically confirmed by accentuated tooth mobility [4]. In our case of several tooth-like structures found externally to the dental arch (Fig 4) in a historical female skull, electron microscopy also showed that they possessed attributes of dental tissue (enamel presence), which confirmed their relation to the dentition and supported the odontogenic potential of the vestibular areas, which can be awakened under pathological condition.

It has been shown that Sox2 is associated with supernumerary tooth formation in odontoma-like tumors induced by Wnt signal activation in mice. The *Sox2* expression in these ameloblastomas has been related to their suggested origin in the *Sox2*-expressing dental lamina epithelium [24]. Sox2 has been detected in the dental epithelium but has also been found in the adjacent oral and vestibular epithelium (shown but not discussed in [25]). Sox2 has been identified as a marker of epithelial stem cells [26], and its expression has also been observed in the dental epithelium [27]. Sox2 is a transcription factor that is essential for maintaining the self-renewal, or pluripotency, of undifferentiated embryonic stem cells. From this aspect and correlated to the present cell-lineage tracing confirming contribution of odontogenic cells to vestibular anlage, the potential of distinct cells to differentiate and form odontogenic tissue may also be maintained in the vestibular area, and if awakened under pathological conditions it can serve as the developmental base for odontogenic pathologies such as odontomas.

## Conclusion

The present results are the first experimental proof of the contribution of dental tissue to the non-dental vestibular epithelium. A common developmental origin of the incisors and oral vestibules in mice as well as in humans allows for the possible re-activation of odontogenic processes under pathological (or experimental) conditions. This fact presents a new view on the non-dental areas of the oral cavity. These areas comprising non-dental but odontogenic tissues could serve as a source of dental stem cells presence, which might represent a new target for researchers in regenerative medicine.

## Supporting Information

S1 Fig Tamoxifen activation of recombination shown in Cre-loxP system (A) compared to actual *Shh* expression visualized by in situ hybridization (B) at ED12.5. Labelled cells documenting the fate of cells expressing *Shh* 24 hours after the tamoxifen injection at ED11.5 in the Cre-loxP system Nr.2 (**A**) and the actual *Shh* expression in WT mouse (**B**) were visualized in the incisor regions (IR) as well as in cheek regions (ChR) of mouse mandibles at ED12.5. Similar patterns of labelled areas in **A** and **B** document that the tamoxifen injection activated the recombination in majority of cells actually expressing *Shh* in this short time window.
(TIFF)

S2 Fig Differences between Cre-loxP system Nr1 and Nr2. The corresponding areas of embryonic heads at ED14.5 (**A**) and 15.5 (**B**) of the same Cre-loxP system Nr. 2 document the fate of the cell population expressing *Shh* at ED11.5. The number of labelled cells (orange arrows) and their localization remain constant; it does not differ substantially in successional stages showing that the labelling of cells is nonrandom. The embryonic lower jaw at ED15.5 (**C**) documents the fate of a whole population of cells expressing *Shh* from the beginning of the prenatal development (Cre-loxP system Nr. 1). The blue labelled cells in **A** and **B** represent a part of a population of blue labelled cells in **C** activated by tamoxifen injection at ED11.5, when *Shh* is expressed in the rudimentary incisor anlage—see also S1 Fig. The blue labelled cells in **A, B, C** have their fate in both: in the incisor germ (black asterisk) as well as in the vestibular anlage (yellow asterisk).
(TIFF)

S3 Fig Tamoxifen activation of recombination at ED14.5. At ED 15.5–24 hours (A) as well as at ED16.5–48 hours (B) after the injection of tamoxifen at ED14.5, only one area of positive labelled cells representing functional incisor (FI) germ is detectable. Semi-serial frontal histological sections (C1-C11) at ED15.5 (24h/tam) document the *Shh* descendant blue cells concentrated to dental epithelium (black asterisk)—in the functional incisor germ. The superficial area descendant of rudimentary incisor anlage as well as the area of the vestibular epithelium (yellow asterisk) remained blue cells negative. It means that the cells expressing *Shh* at ED14.5 and later did not contribute to the vestibular anlage (compare S3 Fig with Fig 3F1–3F8).
(TIFF)
