# Supplementary figures and images for: One Odontogenic Cell-Population Contributes to the Development of the Mouse Incisors and of the Oral Vestibule
Source: PLoS One. 2016 Sep 9;11(9):e0162523. doi: 10.1371/journal.pone.0162523 (PMC5017683; doi:10.1371/journal.pone.0162523)

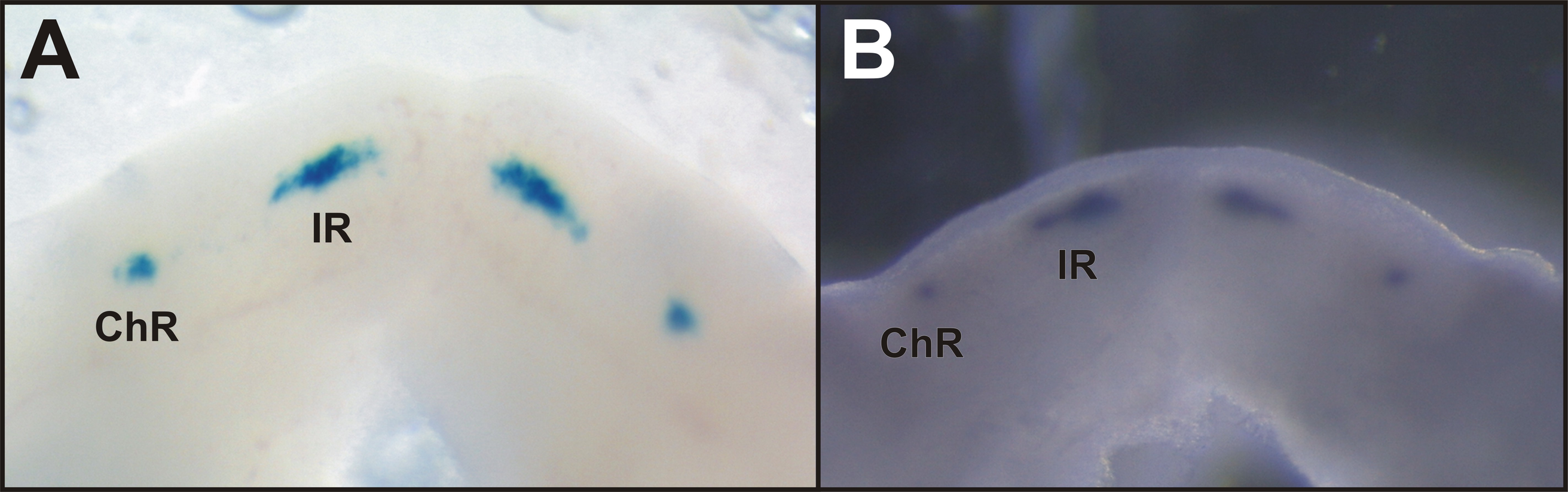

Supplement: S1 Fig — Labelled cells documenting the fate of cells expressing Shh 24 hours after the tamoxifen injection at ED11.5 in the Cre-loxP system Nr.2 (A) and the actual Shh expression in WT mouse (B) were visualized in the incisor regions (IR) as well as in cheek regions (ChR) of mouse mandibles at ED12.5. Similar patterns of labelled areas in A and B document that the tamoxifen injection activated the recombination in majority of cells actually expressing Shh in this short time window. (TIFF) [file pone.0162523.s001.tiff]

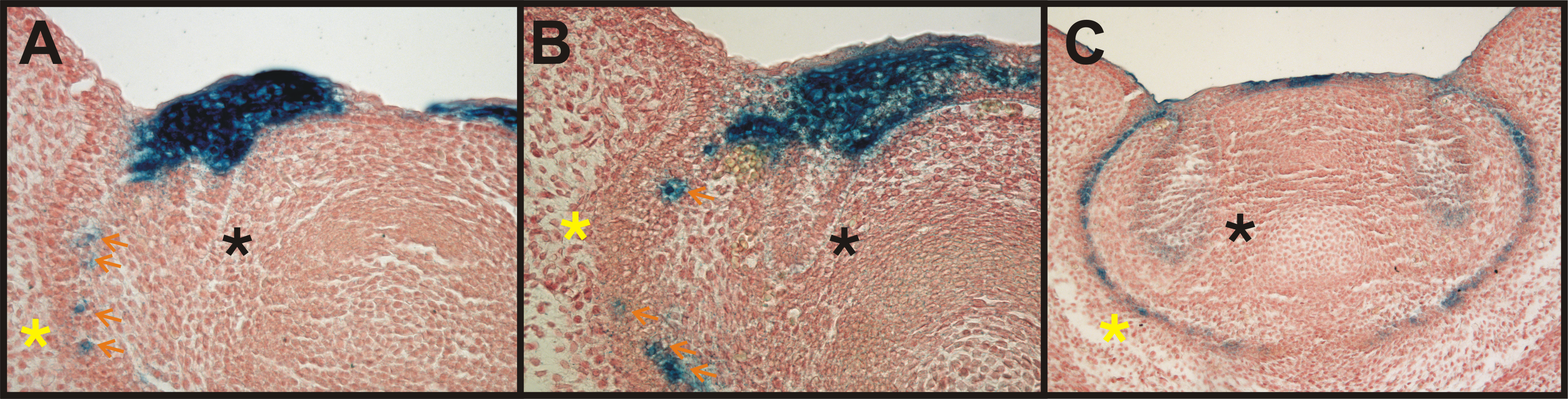

Supplement: S2 Fig — The corresponding areas of embryonic heads at ED14.5 (A) and 15.5 (B) of the same Cre-loxP system Nr. 2 document the fate of the cell population expressing Shh at ED11.5. The number of labelled cells (orange arrows) and their localization remain constant; it does not differ substantially in successional stages showing that the labelling of cells is nonrandom. The embryonic lower jaw at ED15.5 (C) documents the fate of a whole population of cells expressing Shh from the beginning of the prenatal development (Cre-loxP system Nr. 1). The blue labelled cells in A and B represent a part of a population of blue labelled cells in C activated by tamoxifen injection at ED11.5, when Shh is expressed in the rudimentary incisor anlage—see also S1 Fig. The blue labelled cells in A, B, C have their fate in both: in the incisor germ (black asterisk) as well as in the vestibular anlage (yellow asterisk). (TIFF) [file pone.0162523.s002.tiff]

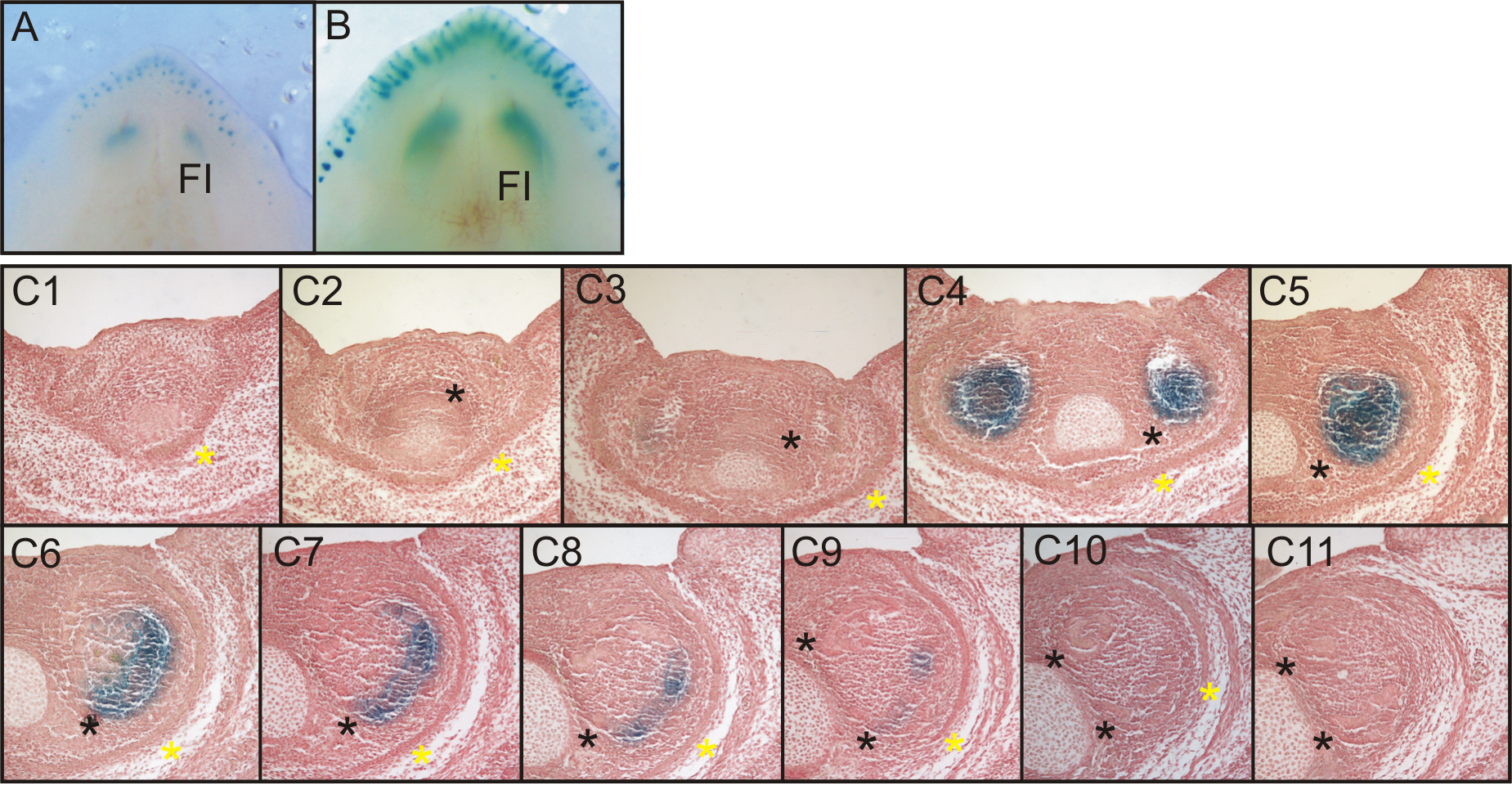

Supplement: S3 Fig — At ED 15.5–24 hours (A) as well as at ED16.5–48 hours (B) after the injection of tamoxifen at ED14.5, only one area of positive labelled cells representing functional incisor (FI) germ is detectable. Semi-serial frontal histological sections (C1-C11) at ED15.5 (24h/tam) document the Shh descendant blue cells concentrated to dental epithelium (black asterisk)—in the functional incisor germ. The superficial area descendant of rudimentary incisor anlage as well as the area of the vestibular epithelium (yellow asterisk) remained blue cells negative. It means that the cells expressing Shh at ED14.5 and later did not contribute to the vestibular anlage (compare S3 Fig with Fig 3F1–3F8). (TIFF) [file pone.0162523.s003.tiff]
